# Comparison of anal cancer screening strategies including standard anoscopy, anal cytology, and HPV genotyping in HIV-positive men who have sex with men

**DOI:** 10.1038/s41416-018-0176-9

**Published:** 2018-07-20

**Authors:** Simon Pernot, Pauline Boucheron, Hélène Péré, Marie-Laure Lucas, David Veyer, Nadia Fathallah, Vincent de Parades, Juliette Pavie, Jeanne Netter, Lio Collias, Julien Taieb, Sophie Grabar, Laurence Weiss

**Affiliations:** 1 Department of Hepato-Gastroenterology and Digestive Oncology, Georges Pompidou European Hospital, APHP, Paris, France; 2 Université Paris Descartes, Sorbonne Paris Cité, Paris, France; 3 Department of Biostatistics and Epidemiology, Cochin-Hôtel Dieu Hospital, APHP, Paris, France; 4 Department of Virology, Georges Pompidou European Hospital, APHP, Paris, France; 5 Department of Clinical Immunology, Georges Pompidou European Hospital, APHP, Paris, France; 6 Department of Proctology, Saint-Joseph Hospital, Paris, France

**Keywords:** Cancer prevention, Population screening

## Abstract

**Background:**

There is no consensus on screening strategy of high-grade intraepithelial neoplasia (HGAIN). Guidelines range from clinical examination with digital anorectal examination followed by standard anoscopy (SA), to anal cytology (Pap)+/− HPV genotyping. We compared screening strategy yields based on Pap, SA, and HPV-16 genotyping alone or in combination in HIV-MSM.

**Methods:**

Pap, SA, and HPV-16 genotyping were performed in all HIV-MSM attending a first anal cancer screening consultation in Paris, France. High-resolution anoscopy, the gold standard to detect HGAIN, was performed in the case of HPV-16 positivity or abnormal cytology. Yield was defined as the number of patients with HGAIN relative to the total number of patients screened.

**Results:**

On 212 patients, the complete strategy (SA + Pap + HPV genotyping) yield (12.7%) was significantly higher than that of SA (3.3%, *p* < 0.001) and HPV-16 alone (6.6%, *p* < 0.05). Although none of the other strategies were significantly different from the complete strategy, Pap + HPV-16 and Pap + SA had closer yields (about 11%), with OR = 0.83 (95% CI [0.44;1.57]) and 0.87 (95% CI [0.46;1.64]), respectively.

**Conclusions:**

Pap combined with HPV-16 genotyping or SA tended towards higher yields compared to Pap alone, and closer to that of the complete strategy.

## Introduction

The main identified risk factors for squamous cell carcinoma of the anus (SCCA) are human papilloma virus (HPV) infection, being a man who has sex with men (MSM), smoking, and human immunodeficiency virus (HIV) infection.^1^ MSM infected by HIV (HIV-MSM) are the highest risk group, with an 80–100 times higher risk of developing SCCA compared to the general population.^2^

The natural history of SCCA shares analogies with that of cervical cancer: a persistent infection by a high-risk HPV (HR-HPV), mainly HPV-16,^3,4^ the development of anal intraepithelial neoplasia (AIN) of low (LGAIN) and high grade (HGAIN) and potentially cancer. As for detection of cervical lesions, the Papanicolaou smear test (Pap) and HPV genotyping have been used in SCCA screening programs in HIV-MSM and have been suggested as effective to decrease SCCA incidence.^5,6^ However, there is no international consensus on SCCA screening. The most commonly accepted modality is anal Pap,^6–8^ which is recommended by Palefsky et al.^9^ Pap alone or combined with digital anorectal examination (DARE) is currently applied in several guidelines in the US^10–12^ and Europe.^13^ However, anal Pap is of suboptimal sensitivity, which leads to underdiagnosis of lesions. HPV genotyping using polymerase chain reaction (PCR) has also been proposed,^3,4,14^ but was not included in guidelines because of its low specificity for the diagnosis of HGAIN. Some authors have proposed restricting genotyping to HPV-16; other HR-HPV such as HPV-18 may be considered as less relevant due to their lower prevalence in HIV-MSM and lower involvement in HGAIN.^15,16^ Following a positive anal Pap or HPV genotyping, high-resolution anoscopy (HRA), which is the gold standard for diagnosis of AIN, is recommended. Finally, clinical examination, based on a DARE and perianal skin examination at least with or without anoscopy, could detect some lesions. Although DARE alone is recommended in few guidelines,^17^ it is well established that it may detect early cancer but not HGAIN.^18^ DARE combined with a standard anoscopy (SA) is recommended in French guidelines for HIV-MSM.^19^ Even if SA diagnoses HGAIN in some cases,^20^ it may underestimate this diagnosis and its performance as a screening tool has never been studied. All these recommendations are based on expert opinions, as few data are available on the performances of global strategies, including anal Pap, HPV-16 genotyping, and SA each alone or combined in HIV-MSM. Consequently, the US Centers for Disease Control and Prevention (CDC) consider that while screening seems to be useful, more evidence is needed to determine the best screening methods.^21^

The aim of this study was to compare seven screening strategies to detect HGAIN in HIV-MSM, HGAIN definitely confirmed on a biopsy performed during HRA or SA: anal Pap, SA, and HPV-16 genotyping each alone, combination of two of them (Pap + SA, Pap + HPV-16 genotyping, or SA + HPV-16 genotyping), or combination of the three (complete strategy: SA + Pap + HPV-16 genotyping).

## Patients and methods

### Anal cancer screening program

**Fig. 1:**
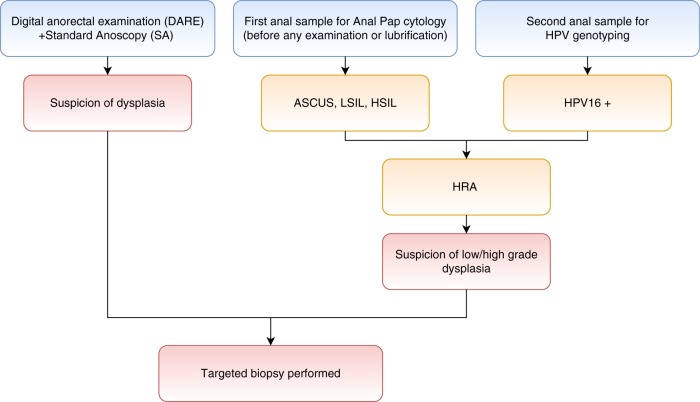
Screening algorithm

An annual anal cancer screening program including Pap, SA, and HPV-16 genotyping was implemented for HIV-MSM followed up in the Department of Clinical Immunology of the Georges Pompidou European Hospital (Paris, France) in 2012. Consecutive patients were enrolled prospectively and had the three procedures referred thereafter as “complete strategy” (Pap, SA, and HPV16 genotyping). HRA was performed in the case of HPV-16 positivity or abnormal cytology (ASCUS, LSIL, HSIL). During SA, if AIN was suspected, a biopsy was performed (Fig. 1). The study was conducted in accordance with the Declaration of Helsinkí. All participants received an information form and informed consent was collected. The use of their medical records for clinical research purposes was approved by the Commission Nationale de l’Informatique et des Libertés (CNIL Authorization No.: 1922081).

### Inclusion criteria

All consecutive patients attending an anal cancer screening consultation for the first time between January 2012 and August 2016 were included in the study. Patients who underwent any prior anal screening test or who were referred with acute anal symptoms, or who had not followed the complete strategy (i.e., complete primary screening and then HRA within 6 months, if indicated) were excluded from the study.

### Screening and diagnostic procedures

Anal Pap, HPV-16 genotyping, and SA were performed during the same consultation. All patients included in the study got the three screening tests.

#### Anal Pap

Anal specimen was collected with polyester fiber swab prior to any examination and lubrification. The swab was then processed using a liquid cytology technique prior to Papanicolaou staining, and finally analysed by a pathologist.

#### HPV genotyping

Anal specimens collected with a brush were removed on UTM medium. During the study, after DNA extraction, HPV-16 detection was performed using INNO-LiPA HPV Genotyping Extra (Innogenetics^®^, Gent) from January 2012 to September 2014 and using a multiplex real-time PCR assay Anyplex^TM^ II HPV28 (Seegene^®^, Seoul) from September 2014 to August 2016.

#### Standard anoscopy

One trained proctologist examined the anal margin and performed DARE and SA with the naked eye. In the event of any abnormality suggestive of AIN, a biopsy was performed. Clinical criteria for suspicion of HGAIN were not precisely defined, and included flat or slightly elevated condyloma, apparent vessels, imprecise borders, induration, or ulceration. HRA was performed in the case of positive screening. HRA is an examination of the squamocolumnar junction, anal canal, and perianal skin, under magnification using a colposcope, with application of 5% acetic acid solution and then Lugol’s iodine. Any suspicious lesion was biopsied under direct visualisation. All HRA were performed by a single trained proctologist who performs around 150 HRA a year, in respect of the IANS International Guidelines.^22^

#### Pathology

Centralised pathology examination was performed by the same experimented gastrointestinal pathologist. AIN were graded according to the Bethesda system, with AIN II and III being classified as HGAIN.^23^

### Statistical analysis

Screening yield was defined as the number of patients with HGAIN relative to the total number of patients screened. Based on the tests results, we were able to know how many HGAIN would have been detected if only anal pap (pap alone) or only SA (SA alone) or HPV-16 genotyping (HPV-16 genotyping alone), or combination of screening tests (Pap + SA, etc.) would have been performed, defining the yield of each strategy. In the main analysis, each strategy yield was compared with that of the complete strategy (i.e., SA combined with anal Pap and HPV-16 genotyping), and anal Pap alone. Altogether, seven strategies were tested: each exam alone, all combinations of two exams in comparison to the complete strategy including the three screening methods. The odds ratio and 95% confidence intervals were estimated.

All tests used were two-tailed and results were considered significantly different if *p*-value < 0.05. All statistical analyses were performed using R software.

## Results

### Study population

Of 253 HIV-MSM who attended a first SCCA screening consultation between 2012 and 2016, 212 (83.8%) fulfilled the inclusion criteria and 41 were excluded, mostly because they did not attend the HRA within 6 months after the screening (*n* = 34). Patient characteristics are described in Table 1. Median age was 51 years and median duration of known HIV infection 15 years. CD4 cell count was above 500/mm^3^ in 73.8% of patients and HIV RNA was below 20 copies/mL in 84%. Baseline characteristics of excluded patients did not differ from those of the study population.

**Table 1:** Patient characteristics (*N* = 212 HIV-MSM)

Patient characteristics	
Age (years): median (IQR)	51 (45–57)
Time since HIV diagnosis (years): median (IQR)	15.2 (5.5–22.6)
Current antiretroviral treatment: yes *N* (%)	209 (98.6)
CD4 cell count (/mm^3^): median (IQR)	682 (491–890)
CD4 nadir (/mm^3^): median (IQR)	271 (153–377)
HIV-RNA levels < 20 copies/mL: *N* (%)	173 (84.0)
Smoking history	
Current smoker *N* (%)	62 (29.2)
Past smoker *N* (%)	44 (20.7)
No smoking history (%)	100 (47.1)
ND (%)	6 (28.3)

### Results of the screening strategies

**Fig. 2:**
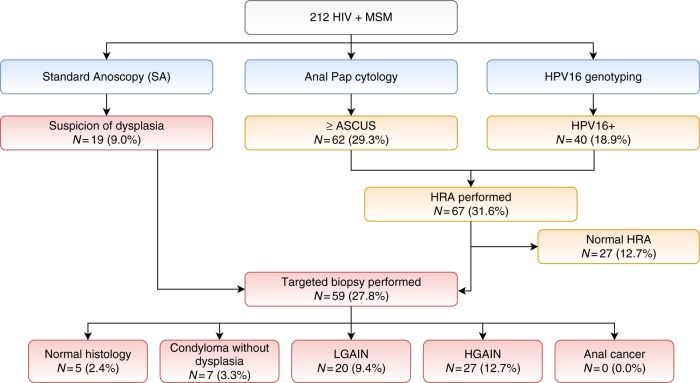
Results of the complete strategy SA + Pap + HPV-16 genotyping

None of the three screening tests was positive in 126 patients (59.4%). Pap was positive in 62 (29.2%) patients, HPV-16 genotyping in 40 (18.9%) patients, and SA in 19 (8.9%) patients. Overall, 86 (40.5%) patients had at least one positive test. Among them, 67 had no macroscopic suspicion of dysplasia and were referred for HRA. Finally, HGAIN was diagnosed by histology in 27 patients (12.7%), including seven with macroscopic lesions at SA (Fig. 2).

The HGAIN detection yields of SA (3.3%) and HPV-16 alone (6.6%) were significantly lower than that of the complete strategy (12.7%) (Table 2). When compared to the complete strategy, dual strategies including anal Pap + HPV-16 genotyping and anal Pap + SA had similar HGAIN detection yields (respectively 10.9%, OR = 0.83 [0.44;1.57] *p* = 0.65 and 11.3%, OR = 0.87 [0.46;1.64] *p* = 0.77). Conversely, although not significantly different from the complete strategy, anal Pap alone and SA + HPV-16 genotyping had slightly lower HGAIN detection yield (both 9%; 0.67 [0.34;1.30] *p* = 0.28).

**Table 2:** HGAIN detection yields of screening strategies compared to the complete and Pap alone strategies

Anal cancer screening strategy (*N* = 212)	Positive screening test	HRA performed	Number of biopsies performed	HGAIN *N* (%)	Strategy vs complete strategy OR (95% CI)	Strategy vs Pap alone OR (95% CI)
SA	19	0	19	7 (3.3%)	0.23 [0.08;0.57] *p* < 0.001	0.35 [0.12;0.89] *p* = 0.02
HPV-16 genotyping	40	39	26	14 (6.6%)	0.48 [0.23;0.99] *p* < 0.05	0.72 [0.32;1.56] *p* = 0.47
Pap	62	59	40	19 (9.0%)	0.67 [0.34;1.30] *p* = 0.27	Ref.
SA + HPV-16 genotyping	53	33	40	19 (9.0%)	0.67 [0.34;1.30] *p* = 0.28	1.00 [0.48;2.06] *p* = 1.00
Pap + HPV-16 genotyping	75	75	48	23 (10.9%)	0.83 [0.44;1.57] *p* = 0.65	1.24 [0.62;2.48] *p* = 0.63
SA + Pap	70	51	52	24 (11.3%)	0.87 [0.46;1.64] *p* = 0.77	1.30 [0.66; 2.59] *p* = 0.52
SA + Pap + HPV-16 genotyping	86	67	59	27 (12.7%)	Ref.	1.48 [0.76;2.92] *p* = 0.27

CI confidence interval, *OR* odds ratio, *SA* standard anoscopy

When strategies were compared to anal Pap alone, the detection yield with SA alone was significantly lower (respectively 3.3% vs 9%). Although none of the yields of other strategies was significantly different from Pap alone, all bimodal strategies adding HPV-16 genotyping or SA to anal Pap, had a higher HGAIN detection yield than Pap alone, except for the combination of SA + HPV-16 genotyping. Finally, the yield with SA was significantly lower than the yields with all other strategies (alone or combined), except for HPV-16 genotyping (Supplementary Table 1).

## Discussion

In the present study of HIV-MSM attending an anal cancer screening for the first time, the prevalence of HGAIN was 12.7%. Among single screening modalities, anal Pap alone had the highest rate of HGAIN detection (9%) compared to SA alone (3.3%) or HPV-16 genotyping alone (6.6%). However, Pap combined with HPV-16 genotyping or with SA gave a trend towards higher yields (about 11%) in comparison to Pap alone or to SA + HPV-16 genotyping (9%).

HRA is the gold standard for diagnosis of HGAIN.^24^ However, most authors agree that HRA cannot be used as a screening test^5,9,14,22,25^ because of its poor acceptability and accessibility. Thus, as in cervical pathology with colposcopy, HRA can only be used as a second-line diagnostic test for anal lesions after positive primary screening.

There is no international consensus on SCCA screening strategy. Pap is currently the most accepted screening test for HGAIN. The sensitivity of anal Pap ranges from 47 to 70% for the detection of AIN of any grade, but seems higher in HIV-MSM,^7,26,27^ reaching 89.2% for HGAIN detection in a recent study by Burgos et al.^28^ In our study, anal Pap alone detected only 19 of 27 (70%) cases of HGAIN, which is, however, more than SA or HPV16 genotyping alone (respectively, 7 and 14 cases).

For the detection of cervical cancers, the use of primary HPV-based screening rather than Pap is endorsed by several European guidelines, based on World Health Organization (WHO) recommendations.^29^ Its use with anal specimens has been proposed.^14^ In recent prospective studies, anal HPV genotyping had a sensitivity of more than 90% in HIV-infected and HIV-uninfected MSM when all high-risk HPV were considered. However, specificity was very low (15–25%) because of the high prevalence of multiple HR-HPV infections in MSM.^28,30^ To increase HPV genotyping specificity, some authors have proposed restricting HRA referral to patients with positive HPV-16 only, based on the high involvement of HPV-16 in HGAIN and SCCA.^15,31,32^ In a multicentre study in HIV-infected men, the specificity of HPV-16 genotyping for the presence of HGAIN was 84%, but its sensitivity was only 35%^31^. In the present study, HPV-16 was found in 40/212 patients (18.9%); 14 of them had HGAIN. If HPV-16 alone was considered, 13/27 HGAIN cases would not have been detected. HPV-16 genotyping alone was significantly inferior to the complete strategy (OR = 0.48; [0.23; 0.99]). Our data clearly indicate that HPV-16 genotyping should not be used as a screening method on its own. However, it would be interesting to consider each HR-HPV genotype alone or in combination as a way to improve the performance of HPV testing. Burgos et al. have shown that the sensitivity and specificity of detection of HPV-16 and/or 18 in predicting HGAIN were only 56% and 63%, respectively.^26^ Other HR-HPV seem more prevalent than HPV-18 in HIV-MSM^15,16^ and it has recently been confirmed that being infected with two or more HR-HPV genotypes was correlated with HGAIN recurrence.^33^ This reinforces the potential interest of focusing on combinations of selected HR-HPV genotypes. Therefore, further investigations are needed to consider the implementation of HR-HPV genotypes other than HPV-16 in anal cancer screening.

In France, national guidelines recommend annual SA for SCCA screening in HIV-MSM.^19^ However, it is widely accepted that a clinical examination with SA including DARE, even performed by a specialist, may miss HGAIN because cases are mostly subclinical and detected only by HRA. In a study of 441 HIV-MSM, DARE failed to detect any of the 156 cases of HGAIN.^18^ In a prospective series of 121 HIV-infected patients, SA was normal in 75% of cases of HGAIN.^8^ In our study, SA detected only 7 of 27 HGAIN cases (26%).

Overall, considering our findings and the literature, anal Pap seems better than SA or HPV16 genotyping, but may be considered as suboptimal in detecting HGAIN if performed alone.

We found that adding SA to anal Pap slightly increased the rate of HGAIN detection (24/27 HGAIN, 89% compared to 19/27). Considering that SA is a simple tool, with a limited increase in costs, it may be implemented as part of the screening strategy. In a recent report by Burgos et al., HR-HPV genotyping was combined with anal cytology. This screening strategy identified almost all HGAIN lesions, but required HRA referral of almost 90% of patients, due to the lack of specificity of global HR-HPV testing.^28^ In our study, compared to Pap alone, there was a trend towards a higher HGAIN detection rate when combining Pap with HPV-16 genotyping (23/27 HGAIN, 85%) and only 75/212 (35%) patients would have required HRA.

Acceptability by the patient and the physician is a major concern for the success of a screening program. We and others have shown that more than 50% of HIV-MSM had never undergone any anal screening test, despite recommendations. Among explanations, lack of information by physicians and lack of time/motivation were highlighted.^34^ Therefore, it is likely that a screening strategy allows self-sampling would be more acceptable. Anal Pap self-sampling has been shown to be acceptable and efficient as a screening strategy,^35^ whereas HPV genotyping self-sampling is currently being studied.^36^

Finally, the interest of screening HGAIN is still unresolved as it has never been shown that such screening could lead to a decrease in cancer incidence, morbidity, or mortality. The natural history of anal cancer is still an unresolved issue. Progression from HGAIN to cancer is not known, and probably lower than in cervical neoplasia. Based on a meta-analysis, Machalek et al. report a theoretical rate of progression of high grade anal dysplastic lesions (or HSIL) of 1/377 (0.27%) among HIV-MSM and 1/4 196 (0.024%) among HIV-uninfected MSM.^37^ Moreover, regression of HGAIN have been described either spontaneously or with immune restoration under ART.^38,39^

There is a need for prospective longitudinal and intervention studies to assess the interest of treating HGAIN in preventing the occurrence of cancer. Indeed, studies evaluating therapeutic intervention on HGAIN were mostly retrospective, not controlled, or too small.^40^ Only one small randomised trial had tested Imiquimod versus placebo in 64 patients.^41^ However, the primary outcome in this study was the regression of HGAIN; progression to cancer was not assessed. Nevertheless, as for detection of cervical lesions, screening programs in HIV-MSM have been suggested as being effective to decrease SCCA incidence.^5,6^ The ongoing ANCHOR study (NCT02135419), currently recruiting more than 5000 HIV patients with HGAIN, randomised into two arms: experimental arm (topical or ablative treatment of lesions) or monitoring every 6 months, will provide a better understanding of the natural history of HGAIN and relevance of treatment.

Our study had some limitations. Firstly, it was not designed to assess the sensitivity and specificity of each strategy, as HRA was only performed in patients with a positive screening test. Secondly, 16.2% of patients (*N* = 41/253) were excluded from the study because they did not adhere to the complete screening strategy. Thirdly, we found a lower prevalence of HGAIN than the 30–42% reported.^31,42^ The prevalence may have been underestimated because of exclusion of patients with symptoms or with any history of previous screening and/or by the design of the study. And we cannot exclude that some patients with negative screening tests had HGAIN, as HRA was not performed in all patients. However, the low prevalence is consistent with the prevalence observed in other recent French cohorts^39,43^ and mirrors a trend in HGAIN epidemiology in France. This low prevalence and the relatively small study population may have underpowered the study. Finally, ours was a single-centre study and the reproducibility and external validity of our results should be confirmed.

In conclusion, among the single screening strategies, anal Pap alone had a higher HGAIN detection yield than SA and HPV-16 genotyping. Among the dual combination strategies, anal Pap + HPV-16 and SA + anal Pap had detection yields similar to that of the complete strategy. However, even though SA decreased the number of HRA performed, it is likely that it might also affect participation or acceptance. In the perspective of self-sampling, anal Pap + HPV-16 genotyping might be the best strategy to increase screening acceptance and to identify HGAIN in HIV-MSM, although it would increase the need for HRA. However, the added value of screening using detection of HPV-16 combined with other selected HR-HPV remains to be assessed in further studies. In addition, extending HRA accessibility is a remaining challenge that needs to be addressed. Finally, these strategies should be evaluated in further studies in the context of low HRA accessibility and health economics systems, with regard to acceptability by the patients.

## Electronic supplementary material

Supplementary Table 1

## Availability of data and material

Data are available at the Department of Biostatistics and Epidemiology, Cochin-Hôtel Dieu Hospital, APHP, Paris, France.
