# Supplementary material for: Comparison of anal cancer screening strategies including standard anoscopy, anal cytology, and HPV genotyping in HIV-positive men who have sex with men
Source: Br J Cancer. 2018 Jul 20;119(3):381–6. doi: 10.1038/s41416-018-0176-9 (PMC6068120; doi:10.1038/s41416-018-0176-9)
Supplement: Supplementary file 1 — Supplementary Table 1 [file 41416_2018_176_MOESM1_ESM.docx]

**Supplementary Table 1:** Comparison of detection rate with the different strategies.
Odds ratio with [95% confidence intervals] and p-value

| **Screening strategy** | **HPV-16 genotyping** | **Pap** | **SA + HPV-16 genotyping** | **Pap + HPV-16 genotyping** | **SA + Pap** | **SA + Pap + HPV-16 genotyping** |
| --- | --- | --- | --- | --- | --- | --- |
| **SA** | 0.48 [0.16;1.31]  p=0.17 | 0.35 [0.12;0.89] p=0.02 | 0.35 [0.12;0.89] p=0.02 | 0.28 [0.10;0.70]  p=0.004 | 0.27 [0.09;0.66]  p=0.002 | 0.23 [0.08;0.57]  p<0.001 |
| **HPV-16 genotyping** | Ref | 0.72 [0.32;1.56] p=0.47 | 0.72 [0.32;1.56] p=0.47 | 0.58 [0.27;1.22]  p=0.17 | 0.55 [0.26;1.15]  p=0.12 | 0.48 [0.23;0.99]  P<0.05 |
| **Pap** |  | Ref | 1.00 [0.48;2.06] p=1.00 | 0.81 [0.40;1.61]  p=0.63 | 0.77 [0.38;1.52]  p=0.52 | 0.67 [0.34;1.30]  p=0.27 |
| **SA + HPV-16 genotyping** |  |  | Ref | 0.81 [0.40;1.61]  p=0.63 | 0.77 [0.38;1.52]  p=0.53 | 0.67 [0.34;1.30]  p=0.28 |
| **Pap + HPV-16 genotyping** |  |  |  | Ref | 0.95 [0.49;1.83]  p=1.00 | 0.83 [0.44;1.57]  p=0.65 |
| **SA + Pap** |  |  |  |  | Ref | 0.87 [0.46;1.64]  p=0.77 |
| **SA + Pap + HPV-16 genotyping** |  |  |  |  |  | Ref |

SA: Standard anoscopy. Each cell of the table shows the odds ratio with [95% confidence intervals] and p-value comparing the strategy in columns vs the strategy in rows.
